# Median arcuate ligament syndrome presenting as acute mesenteric hemorrhage: A case report

**DOI:** 10.1016/j.radcr.2025.09.008

**Published:** 2025-09-24

**Authors:** Salim Gnabode, Dina Medhani Asfaha, Travis E. Meyer, Sharon Siegel

**Affiliations:** a Department of Radiology, Maine Medical Center, Portland, ME, USA; b Interventional Radiology, Maine Medical Center, Portland, ME, USA; c Tufts University School of Medicine, Boston, MA, USA

**Keywords:** Median arcuate ligament syndrome, Celiac artery stenosis, Arc of Bühler pseudoaneurysm, Pancreatoduodenal artery pseudoaneurysm, Acute mesenteric hemorrhage, Emergency radiology

## Abstract

Median arcuate ligament syndrome (MALS) is a relatively common, yet under-recognized condition caused by external compression of the celiac artery by the median arcuate ligament. Although often asymptomatic, MALS may present with chronic gastrointestinal symptoms, and rarely, with acute complications due to collateral vessel rupture.

We present the case of a 59-year-old woman who arrived at a rural emergency department with acute left flank pain and emesis, ultimately diagnosed with intra-abdominal hemorrhage from a ruptured pseudoaneurysm. In chronic celiac artery stenosis, collateral pathways, particularly the Arc of Bühler and the pancreaticoduodenal arcade can become hypertrophied and predisposed to pseudoaneurysm formation. Rupture of these pseudoaneurysms may mimic other causes of acute mesenteric bleeding, such as hemorrhagic pancreatitis, and carry high mortality if not promptly identified.

This case underscores the importance of recognizing splanchnic arterial collaterals as potential sources of hemorrhage in MALS and highlights the need for early imaging, especially in resource-limited settings. Prompt angiographic assessment and embolization can be lifesaving.

## Case report

A 59-year-old woman with a past surgical history of gastric bypass and hernia repair (11 years prior), and hysterectomy (15 years prior), presented to a rural emergency department from an urgent care clinic with sudden-onset, sharp left flank pain accompanied by nausea and vomiting. She reported being in her usual state of health prior to symptom onset. The patient denied any recent trauma, history of coagulopathy, or use of anticoagulants. She reported drinking 3 glasses of wine several times per week. On examination, the patient was in acute distress, writhing on the stretcher with left flank pain and emesis. She was hypothermic (34.6°C), tachypneic (respiratory rate: 44 breaths/min), and hypertensive (blood pressure: 180/94 mmHg). Abdominal examination revealed tenderness localized to the epigastric and left upper quadrant regions. Laboratory results showed leukocytosis (WBC: 13,000/µL) with a left shift (90% neutrophils), hemoglobin of 12.8 g/dL, hematocrit of 38.4%, and lactate of 2 mmol/L. Intravenous morphine provided pain relief.

**Fig. 1:**
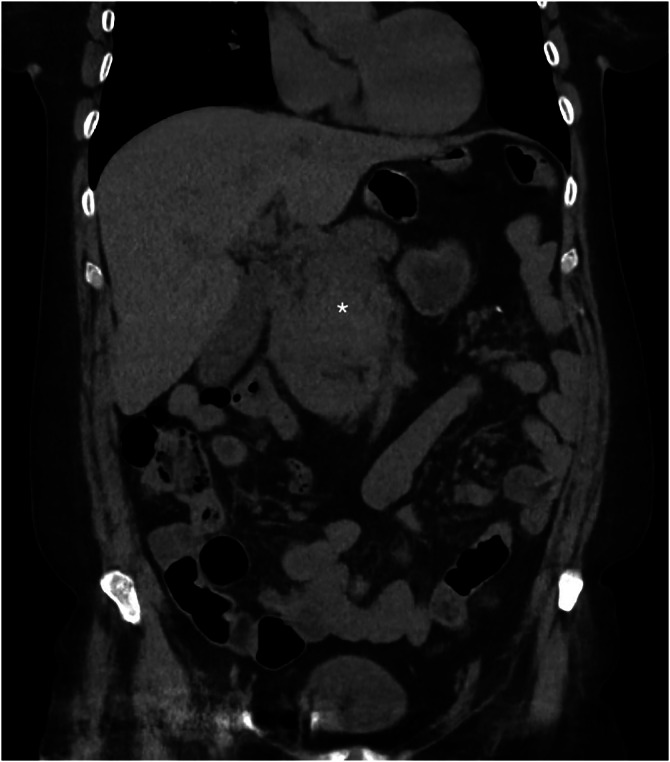
Non-contrast CT abdomen pelvis shows thickening of the pancreatic body (asterisk) with peripancreatic inflammatory changes.

**Fig. 2:**
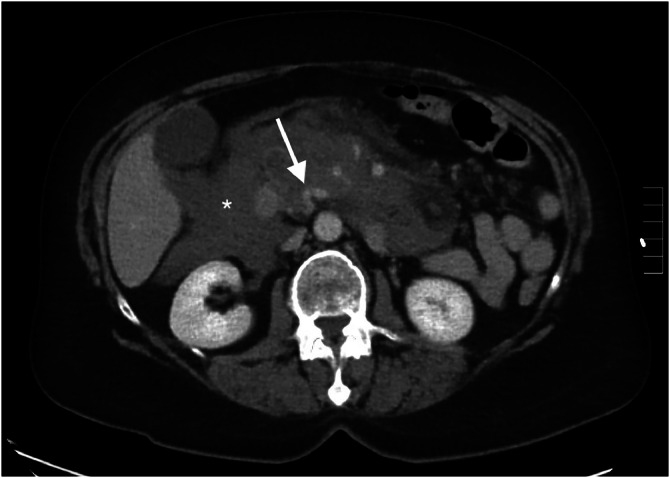
Portal venous phase CT of the abdomen and pelvis reveals acute mesenteric hemorrhage (asterisk) with formation of prominent, serpiginous splanchnic collateral vessels (arrow).

Given concern for obstructive uropathy by the rural emergency physician, a non-contrast CT of the abdomen and pelvis was obtained (Fig. 1). The differential diagnosis also included pancreatitis and bowel obstruction. The CT was negative for obstructive uropathy but demonstrated thickening of the pancreatic body and peripancreatic inflammation, concerning for pancreatitis; however, an underlying neoplasm could not be excluded. Lipase was within normal limits (31 U/L). Urinalysis showed no leukocyte esterase or pyuria but did reveal trace microscopic hematuria, ketonuria, and proteinuria. Due to inconclusive findings and ongoing concern for underlying pathology, a contrast-enhanced CT of the abdomen and pelvis was obtained after consultation with the rural emergency radiologist. This study (Fig. 2) demonstrated extensive acute mesenteric hemorrhage, which had significantly progressed over 90 minutes, and was concerning for hemorrhagic pancreatitis, although lipase was normal.

The rural emergency radiologist consulted a second radiologist at our facility for further interpretation. Upon review of prior imaging, including a CT angiography (CTA) of the abdomen and pelvis performed 6 years earlier, the second radiologist identified celiac artery stenosis secondary to median arcuate ligament compression, which was not reported in the prior CTA. Over the intervening years, the stenosis had significantly progressed, accompanied by the development of prominent splanchnic collateral vessels. The acute mesenteric hemorrhage was ultimately attributed to rupture of a visceral pseudoaneurysm.

**Fig. 3:**
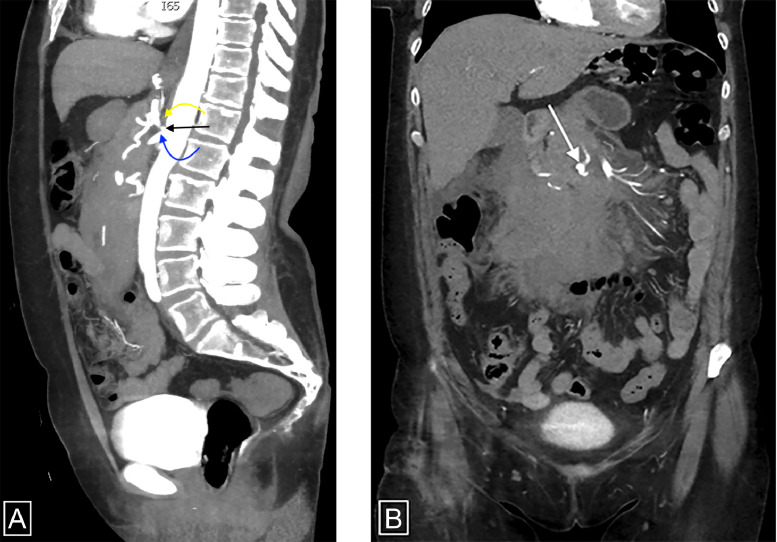
(A) Sagittal CT angiography of the abdomen and pelvis reveals compression of the celiac artery (black arrow) by the median arcuate ligament (curved yellow arrow), resulting in characteristic narrowing with a “hooked” appearance. The superior mesenteric artery (SMA) is shown (curved blue arrow) (B) Coronal CT angiography of the abdomen and pelvis reveals 6mm Arc of Bühler pseudoaneurysm (white arrow) surrounding by extensive acute mesenteric hemorrhage.

Repeat laboratory evaluation revealed a hemoglobin of 10.8 g/dL and hematocrit of 34.1%, down from 12.8 g/dL and 38.4% four hours earlier. Following consultation with a rural general surgeon, the patient was transferred via life flight to our tertiary care center for further evaluation. She remained hemodynamically stable during transport. At our facility, the patient continued to report mild abdominal discomfort. Vital signs included a blood pressure of 152/90 mmHg, heart rate of 82 bpm, and respiratory rate of 14 breaths/min. She was admitted to the emergency general surgery service. A CTA of the abdomen and pelvis was obtained to evaluate the mesenteric vasculature and assess for active extravasation (Fig. 3). This revealed extensive intra-abdominal hemorrhage, which had progressed significantly over 3 hours, without evidence of active contrast extravasation. Chronic celiac artery stenosis due to median arcuate ligament compression was noted, along with two 6 mm pseudoaneurysms—one in the hypertrophied posterior inferior pancreaticoduodenal artery and one in the hypertrophied Arc of Bühler.

**Fig. 4:**
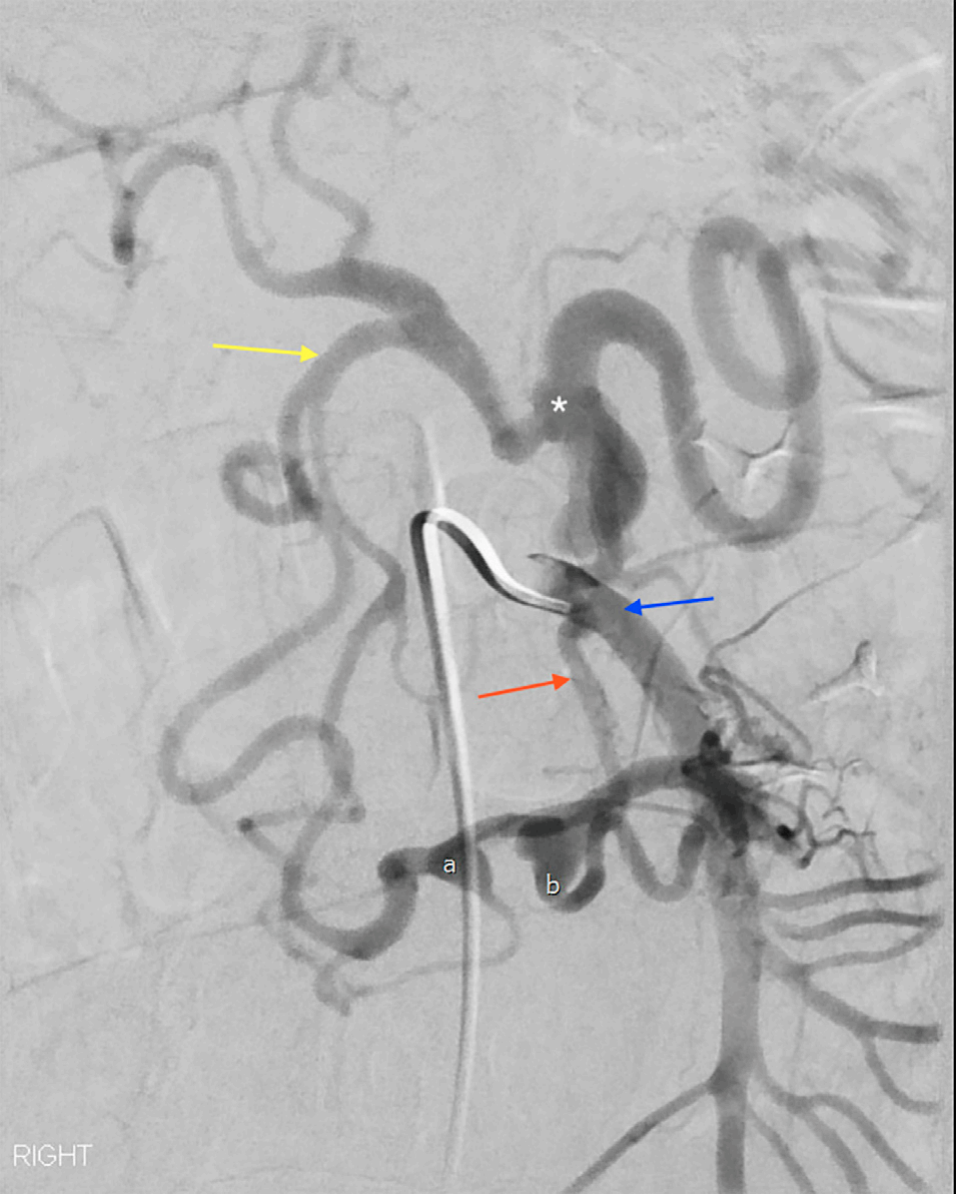
Catheterization of the SMA (blue arrow) was performed during angiography. A pseudoaneurysm is identified in the posterior inferior pancreatoduodenal artery (A), as well as the Arc of Bühler (B). The Arc of Bühler is an anatomic variant that connects the SMA (blue) to the celiac artery (asterisk). The gastroduodenal artery is also shown (yellow arrow).

Interventional radiology was consulted for emergent angiography. The mesenteric arterial system was accessed using a 5 French SOS catheter, followed by a 5 French C2 Glide catheter and a 2.4 French Progreat microcatheter. Angiography (Fig. 4) confirmed severe (99%) focal stenosis at the origin of the celiac artery, consistent with median arcuate ligament syndrome (MALS). There was hypertrophy, flow reversal, and pseudoaneurysm formation involving both the posterior inferior pancreaticoduodenal artery and the Arc of Bühler. The Arc of Bühler pseudoaneurysm was identified as the source of bleeding and was successfully embolized. The patient tolerated the procedure and selective coil embolization without immediate complications. Post-procedure, she developed signs of paralytic ileus, which improved with supportive care and the gradual return of bowel function. Her diet was advanced as tolerated. On post-procedural day 2, her hemoglobin dropped to 6.7 g/dL, likely due to hemodilution and equilibration rather than ongoing hemorrhage, as there were no signs of hemodynamic instability or worsening pain. She received one unit of packed red blood cells with an appropriate response, and hemoglobin levels stabilized. The patient was discharged home on post-procedural day 3 in stable condition, with outpatient follow-up arranged with her primary care physician and vascular surgery. A median arcuate ligament release was planned following her recovery.

## Discussion

MALS, also known as celiac artery compression syndrome, is caused by external compression of the celiac artery by the median arcuate ligament of the diaphragm. First described by Harjola in 1963 [1], it remains a controversial diagnosis due to the high prevalence of asymptomatic celiac artery compression [2]. Compression severity varies with respiration, typically worsening during expiration, thus making diagnosis challenging and dependent on appropriately timed imaging [3].

While MALS classically presents with chronic symptoms such as postprandial abdominal pain, nausea, vomiting, and weight loss [2,4], acute presentations can occur in the setting of collateral vessel complications. Chronic celiac artery stenosis can lead to the development of collateral circulation in some patients, via the pancreaticoduodenal arcade, dorsal pancreatic artery, and arc of Bühler to maintain visceral perfusion [5,6]. The arc of Bühler, a persistent embryonic anastomosis between the superior mesenteric artery (SMA) and celiac artery, is present in a minority of individuals and infrequently implicated in mesenteric hemorrhage. In contrast, the pancreaticoduodenal arcade is a well-established arterial network that provides communication between the celiac axis and SMA through the superior and inferior pancreaticoduodenal arteries. Increased flow through these collateral pathways, particularly under conditions of hemodynamic stress, can predispose to pseudoaneurysm formation and eventual rupture, resulting in acute mesenteric hemorrhage.

In this case, the patient presented to a rural emergency department with vague but concerning abdominal symptoms. Initial non-contrast CT (Fig. 1) demonstrated thickening of the pancreatic body and peripancreatic inflammatory changes, raising suspicion for pancreatitis or an underlying neoplasm. As symptoms progressed, accompanied by a decline in hemoglobin and new findings of acute mesenteric hemorrhage on contrast-enhanced CT (Fig. 2), the working diagnosis shifted toward hemorrhagic pancreatitis. However, a normal lipase level, combined with a subsequent review of the case and a CT angiography (CTA) from 6 years earlier by a second radiologist, revealed previously undiagnosed chronic celiac artery stenosis with extensive peripancreatic collateral vessel development over the intervening years.

These findings prompted further vascular imaging. CTA (Fig. 3) obtained during the patient’s admission demonstrated hallmark features of MALS, including focal celiac artery narrowing with a characteristic “hooked” appearance, post-stenotic dilatation, hypertrophied collateral vessels, and the presence of 2 pseudoaneurysms; one arising from the posterior inferior pancreaticoduodenal artery and another ruptured pseudoaneurysm from the Arc of Bühler. The hooked configuration of the celiac artery is a distinguishing imaging feature that helps differentiate MALS from atherosclerotic disease [2]. Angiography with respiratory maneuvers remains the gold standard for confirming the diagnosis of MALS and evaluating splanchnic pseudoaneurysms, while also enabling therapeutic intervention. In this case, transcatheter embolization was successfully performed.

This case underscores the critical importance of recognizing splanchnic collateral arising from the pancreaticoduodenal arcade and arc of Bühler as indicators of chronic celiac artery stenosis from MALS on contrast-enhanced CT in the emergency setting, particularly in resource-limited environments. Identification of these collaterals by the emergency radiologist should prompt careful evaluation for pseudoaneurysms. This is crucial, as pancreaticoduodenal artery pseudoaneurysms carry a mortality rate of 30%-50% if left untreated [7]. Prophylactic endovascular intervention is therefore recommended in patients with celiac artery stenosis–associated pseudoaneurysms, regardless of size, due to their high rupture risk [7]. While there are no definitive diagnostic or management guidelines for MALS, treatment options include surgical ligament release, celiac ganglionectomy, and vascular revascularization [2,4].

## Ethical approval

This case report was reviewed by the institutional IRB and determined not to constitute human subjects research; IRB approval was not required.

## Patient consent

Written informed consent was obtained from the patient for publication of this case report.
